# Mamba-enhanced codebook learning with anatomical constraints for liver and tumor segmentation in 3D CT volumes

**DOI:** 10.3389/fmedt.2026.1708094

**Published:** 2026-03-18

**Authors:** Yanfei Teng, Xiang Li, Zhenpeng Chen, Shunlin Guo

**Affiliations:** 1 The First Clinical Medical College of Lanzhou University, Lanzhou, China; 2 Center for Medical Artificial Intelligence, Shandong University of Traditional Chinese Medicine, Jinan, China; 3 Department of Radiology, The First Hospital of Lanzhou University, Lanzhou, China

**Keywords:** 3D medicalimage analysis, anatomical prior, codebook quantization, liver and tumor segmentation, mamba, multi-scale feature learning

## Abstract

Precise delineation of the liver and its tumors in 3D CT scans plays a vital role in clinical diagnosis and therapeutic planning. However, current deep learning approaches frequently struggle with tumor heterogeneity, varying lesion sizes, and ambiguous boundaries, which can limit their effectiveness. To address these issues, we propose an end-to-end hierarchical network that effectively integrates multi-scale context modeling, global relational learning, and structured feature representation. First, a multi-scale texture encoder is designed to capture tumor characteristics across different spatial resolutions. To model long-range dependencies across slices, we introduce a global relational representation module built upon the emerging Mamba architecture, enabling efficient and directional context aggregation in 3D volumes. Second, to enhance feature compactness and stability, we propose a learnable codebook module that quantizes high-dimensional features into a finite set of semantic prototypes, promoting discriminative representation learning while suppressing redundancy. Furthermore, anatomical prior knowledge—specifically, the spatial constraint that tumors must reside within the liver—is incorporated via an inclusion loss, which explicitly regularizes the segmentation outputs. Comprehensive experiments on the public LiTS dataset show that our method attains state-of-the-art results, surpassing existing methods in Dice score, volumetric overlap error (VOE), and boundary metrics (ASD and 95HD). Ablation analyses confirm the individual contribution of each module, demonstrating the architecture’s effectiveness for accurate and reliable liver and tumor segmentation.

## 1 Introduction

Liver cancer is a major contributor to cancer-related fatalities worldwide [1]. Hepatocellular carcinoma (HCC), the most common type of primary liver cancer, is the fifth most prevalent cancer and the third highest cause of cancer deaths globally [2]. Early diagnosis and timely intervention are critical for successful tumor resection and improved patient outcomes. Accurate tumor segmentation enables the extraction of quantitative volumetric data and texture features, which play a vital role in liver treatment planning [3]. It also enhances the reliability of treatment response assessment, tumor classification, and prediction of patient survival.

**Figure 1:**
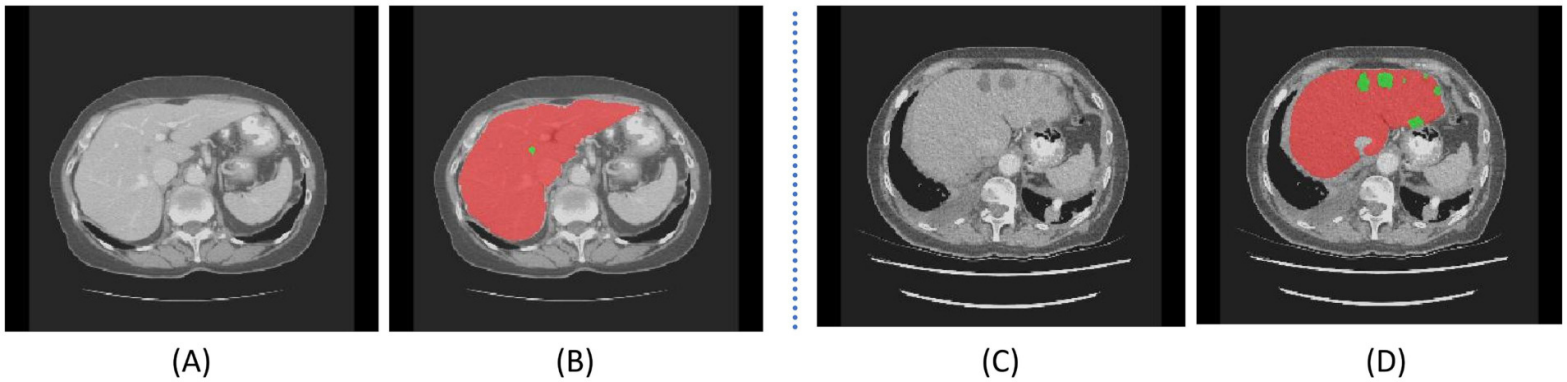
CT scan shows significant changes in the shape, size, and location of liver lesions. **(A)** and **(C)** display CT scan results obtained from individual patients, while **(B)** and **(D)** are labeled results, where green area represents tumor and red represents liver.

Computed tomography (CT) is a mainstream imaging modality for liver lesion measurement, due to its fast acquisition ability and low cost compared to MRI [4]. However, as shown in Figure 1, accurate segmentation of liver lesions in CT images remains challenging due to significant variations in tumor shape, intensity, and position, along with poorly defined margins and the presence of noise—especially from contrast enhancement. Despite these challenges, manual lesion segmentation is still considered the clinical standard for volume measurement, especially for 3D imaging data, although it is labor-intensive, time-consuming, and subject to inter-observer variability.

Since the advent of U-Net [5], the majority of liver lesion segmentation methods have been built upon the U-shaped convolutional neural network (CNN) framework, featuring an encoder for extracting hierarchical features and a decoder for reconstructing the segmentation mask [6–8]. Due to its ability to automatically learn high-level image features and enhance task performance, this method has achieved remarkable success in liver tumor segmentation, exemplified by the top-performing method in the 2017 Liver Tumor Segmentation (LiTS) Challenge [9], which was based on deep learning. While CNNs excel at capturing local contextual feature, their performance is often limited by the inherent locality of convolution operations. This limitation hinders their capacity to capture long-range spatial dependencies, which is essential for accurately segmenting intricate and heterogeneous lesion structures in medical imaging [10].

Transformer has a powerful ability to capture long-range dependencies and has been applied in medical image segmentation, such as [11, 12]. These methods use Transformer blocks to construct encoders or decoders, and implement downsampling and upsampling of feature maps through patch merging and patch extension. Although Transformer based methods can achieve effective remote dependency modeling, they are difficult to train due to the lack of inductive bias [13]. In addition, Transformer requires a large amount of annotated data for training to achieve better performance, which is difficult for medical image segmentation tasks [14].

Recent studies have shown that combining CNN and Transformer can effectively leverage their respective advantages, leading to significant advancements in both natural and medical image segmentation tasks [15–17]. However, there are still several issues and challenges in designing effective hybrid CNN and Transformer models to achieve accurate 3D liver and lesion segmentation.

### 1.1 How to effectively learn CNN and transformer features from 3D CT images

Effective feature learning from 3D CT images using CNNs and Transformers is vital for segmentation. Three key challenges arise: First, liver tumors vary widely in size, shape, and location across patients. Fixed-size convolution kernels have limited receptive fields, restricting them to specific scales and hindering simultaneous segmentation of small and large tumors [18]. Second, 3D volumes are reconstructed from 2D slices with high in-plane resolution but larger through-plane spacing, resulting in anisotropic voxels. This anisotropy challenges standard 3D convolutions, which assume more uniform spatial sampling [19]. Third, 3D liver data is high-dimensional with complex spatiotemporal dependencies. The standard self-attention of Transformer scales quadratically with sequence length, leading to high memory use and slow inference [20].

### 1.2 How to characterize the DOF–redundancy trade-off in features

A key challenge in deep representation learning is balancing expressive capacity and feature redundancy [21]. Effective degrees of freedom (DOF) quantify a model’s capacity to learn diverse and discriminative features, which is essential for capturing complex anatomical variations [22]. Yet, deep networks, especially CNN-Transformer hybrids, often produce redundant features due to channel correlation, spatial smoothness, or over-parameterization, increasing computational cost and impairing generalization through entangled representations [23, 24]. A principled framework to characterize the DOF–redundancy trade-off is still lacking, limiting representation evaluation and efficient architecture design. Addressing this gap would advance regularization, model compression, and interpretability in medical image analysis.

### 1.3 How to introduce anatomical prior into models to improve performance

Anatomical prior refers to known knowledge or regularities about human anatomy [25, 26], such as shape priors (organs have relatively fixed shapes) or spatial relationship priors (organs maintain consistent relative positions). However, in previous liver and tumor segmentation studies, most methods primarily rely on data-driven deep learning models to learn the relationship between image features and corresponding segmentation labels from large amounts of annotated data, with insufficient explicit modeling and effective integration of anatomical priors.

Thus, to address the aforementioned challenges, we propose an end-to-end deep learning model for automatic liver and tumor segmentation. First, to effectively capture features from 3D CT volumes, we design a multi-scale texture encoder to model tumor characteristics at different scales. To better capture relationships across slices in CT volumes, we introduce a global relational representation module based on the Mamba architecture. Second, while the multi-scale texture encoder generates rich feature information, we propose a codebook module to learn more compact and stable representations. This module explicitly constrains the capacity of the feature space by quantizing high-dimensional feature vectors into a limited set of learnable prototype vectors. It promotes feature diversity while suppressing redundant patterns, thereby reducing representation complexity without sacrificing expressive power. Furthermore, to incorporate anatomical prior knowledge, we introduce an inclusion loss into the objective function to enforce the spatial constraint that tumors must reside within the liver, further enhancing segmentation performance.

In summary, our main contributions are:

- We propose a hierarchical network integrating multi-scale texture encoding and global relational modeling, enabling comprehensive feature learning to address tumor heterogeneity, scale variation, and boundary ambiguity in 3D CT.
- A Mamba-based global relational module captures long-range spatial dependencies efficiently and directionally, achieving 3D context modeling with linear complexity.
- A codebook module enforces compact, discriminative representations via feature quantization, while an inclusion loss incorporates anatomical prior by constraining tumors within the liver, improving plausibility and reducing false positives.

## 2 Related work

### 2.1 Liver and tumor segmentation on CT images

Currently, the majority of research efforts are directed toward applying automatic liver tumor segmentation techniques to CT imaging. Given the relatively low cost of CT and its frequent use in preoperative planning, obtaining relevant data is relatively convenient in clinical settings. Early researchers commonly employed traditional computer vision techniques for liver segmentation, including coarse segmentation utilizing thresholding [27–29] and morphological operations [30, 31], as well as refinement through clustering and geometrically deformable models [32, 33]. Alternatively, they utilized level set-based methods for semi-automatic liver segmentation [34]. However, traditional methods are highly sensitive to noise, artifacts, and non-uniformity in CT images, which can easily lead to inaccurate segmentation results. More importantly, conventional approaches depend on handcrafted features—such as intensity, texture, and shape priors—that may fail to fully represent the complex and variable anatomical structures of the liver across different imaging scenarios.

In 2017, Christ et al. [35] pioneered a cascaded approach using two fully convolutional neural networks, followed by 3D dense conditional random field post-processing to refine both liver and lesion segmentation. Vorontsov et al. [33] proposed a single-stage end-to-end trainable model for simultaneous liver and tumor segmentation, eliminating the need for conventional post-processing steps. With the development of U-Net networks, Li et al. [8] introduced a hybrid densely connected U-Net (H-DenseUNet), employing a 2D dense U-Net to capture intra-slice features and leveraging 3D contextual information for hierarchical aggregation of volumetric features. Seo et al. [36] improved the basic U-Net architecture by combining object related advanced features to achieve better liver and tumor segmentation. Alalwan et al. [37] introduced 3D-DenseUNet-569, a highly efficient 3D deep learning architecture for liver and tumor segmentation. This fully 3D semantic segmentation network achieves greater depth while maintaining a reduced number of trainable parameters by utilizing depthwise separable convolutions in place of standard convolutions, thereby improving computational efficiency. Song et al. [38] proposed a network that captures the correlation between 2D and 3D data. In 2024, Wang et al. [39] proposed SBCNet, a dual-branch liver tumor segmentation model featuring a multi-scale adaptive context encoder and a boundary enhancement module that integrates contour learning with the Sobel operator. A hybrid multi-task loss further improves segmentation by jointly optimizing for tumor size and boundary accuracy. Hu et al. [40] proposed MSML-AtUNet, a novel network architecture that utilizes multi-level dilated convolutions to extract multi-scale features, effectively enlarging the receptive field of convolutional kernels to capture more comprehensive contextual information.

### 2.2 Transformer in medical image segmentation

The Transformer architecture has emerged as a powerful alternative to CNNs in medical image segmentation, offering superior capability in modeling long-range spatial dependencies and global contextual information [41]. Unlike CNNs, which are limited by local receptive fields, Transformers leverage self-attention to capture holistic anatomical relationships across distant regions—critical for accurately segmenting irregular or diffuse pathological structures such as tumors and ischemic lesions [42]. Recent advances, including the integration of Transformers with CNN backbones [e.g., in UNETR [43] and TransUNet [44]], have shown notable improvements performance gains on various 2D and 3D medical imaging tasks. However, the direct application of standard Transformers to volumetric medical data remains challenging due to their high computational complexity, memory demands, and weak inductive biases for spatial locality [45, 46]. Research has increasingly focused on designing efficient, domain-adapted Transformer variants that balance modeling power with practical constraints in clinical applications.

## 3 Methods

**Figure 2:**
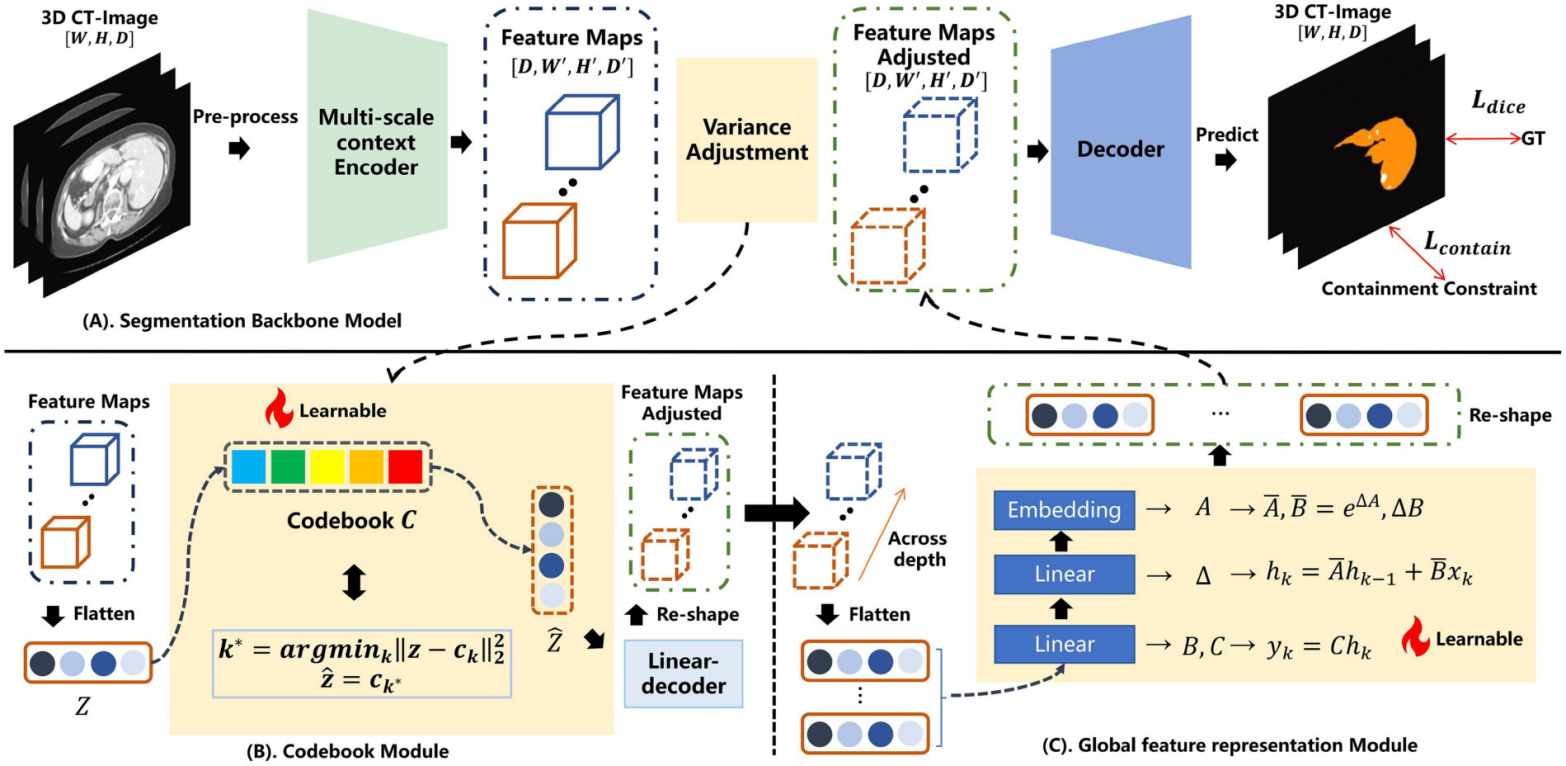
The overall architecture of the proposed method. **(A)** The backbone of the proposed method is displayed, which includes multiple components. **(B)** The codebook module is illustrated. By quantizing high-dimensional feature vectors into a finite set of learnable prototype vectors, it explicitly constrains the capacity of the feature space. **(C)** The global feature representation module was demonstrated, which is used to capture the sequential relationships between longitudinal high-level semantic features and effectively model the relationships between different slices in CT images.

The overall architecture of the proposed model is illustrated in Figure 2. Overall, our proposed model integrates CNN, Mamba, and U-Net to fully leverage the strength of CNN in local feature extraction, the capability of Mamba in modeling long-range dependencies, and the encoder-decoder architecture of U-Net, enabling more comprehensive feature understanding. The backbone of the model is a U-Net–based segmentation architecture (Section 3.1). Specifically, the model consists of a multi-scale context encoder (Section 3.2), a codebook module (Section 3.3), a global representation module (Section 3.4), and an optimized objective function (Section 3.5), achieving comprehensive integration of local and global contextual information and thereby further improving liver tumor segmentation performance.

### 3.1 U-Net-based segmentation backbone

We adopted a 3D U-Net [47] architecture consisting of an multi-scale context encoder, bottleneck, and decoder for volumetric liver and tumor segmentation. The multi-scale context encoder uses 3D convolutional kernels and pooling paths including 3×3×3, 5×5×5, and 7×7×7 to further enhance the capture ability of tumor features at different scales, and uses continuous max pooling to gradually extract hierarchical features. The bottleneck improves the representation ability at the lowest resolution. The decoder used transposed convolutions for up-sampling and concatenated the corresponding encoder features via skip connections, followed by convolutional blocks to refine spatial information. To address potential mismatches between skip connections and up-sampled features, we implemented an automatic center cropping and padding strategy to ensure spatial alignment. Finally, a 1×1×1 convolution layer was applied to map the features into segmentation masks.

### 3.2 Multi-scale context encoder

**Figure 3:**
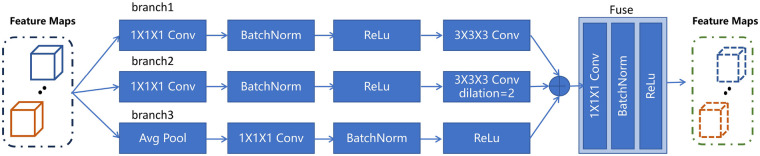
The architecture of the proposed multi-scale contexl encoder. It includes three feature extraction branch structures and fuses the features.

The liver, as the largest internal organ in the human body, typically occupies a large area in CT images and is considered a large-scale structure. In contrast, tumors, especially early-stage or small ones, can be extremely tiny, occupying only a few to dozens of pixels, making them small-scale targets. A large receptive field may overlook the fine details of small tumors, while a small receptive field struggles to capture the global shape and surrounding contextual features of the liver. Additionally, CT images are typically high-resolution 3D volumetric data. Processing full-resolution data directly incurs enormous computational cost. Encoders efficiently capture large-scale information by progressively reducing resolution through downsampling, thereby expanding the receptive field. However, downsampling inevitably leads to the loss of spatial details, which is highly detrimental to the precise localization of small tumors. Therefore, the ability to capture features from macro to micro scales, as well as to preserve or restore critical fine details, is crucial for liver and tumor segmentation. To address the aforementioned challenges, we propose a multi-scale contexl encoder. As illustrated in Figure 3, the encoder consists of three parallel branches designed to capture features at multiple scales, enabling the model to simultaneously perceive both global anatomical structures (e.g., the liver) and fine-grained local details (e.g., small tumors).

Specifically, in the first branch, we employ a combination of 1×1×1 and 3×3×3 convolutions to extract both channel-wise and local spatial features. In the second branch, we utilize a 1×1×1 convolution followed by a 3×3×3 dilated convolution with an appropriate dilation rate to effectively simulate the receptive field of a 5×5×5 convolution, thereby capturing mid-range contextual information without significantly increasing computational cost. In the third branch, we apply average pooling to aggregate global contextual information across the spatial dimensions, followed by a 1×1×1 convolution to adapt the channel dimension and refine the pooled features.

The feature maps from the three branches are then concatenated along the channel axis to construct a rich, multi-scale feature representation. A 1×1×1 convolution is then applied to fuse these multi-scale features, reducing channel redundancy and enhancing feature expressiveness. This fusion strategy allows the network to selectively enhance informative features at different scales, strengthening its capacity to precisely segment both large anatomical regions and small lesions in complex and varying imaging environments.

### 3.3 Codebook module

After passing through the multi-scale contexl encoder, we have captured semantic features of liver and tumor images ranging from macro to micro scales. Although this process yields a rich feature representation, it also introduces feature redundancy, which can negatively impact the generalization ability of network. Thus, to achieve compact feature representation, we designed a CodeBook module that performs semantic compression on high-dimensional 3D features. This forces the model to learn more compact and semantically stronger representations, reducing redundant information, enhancing generalization capability, and preventing overfitting.

As shown in Figure 2, given encoder features $Z\in R^{B\times C\times D\times H\times W}$, we flatten to site-wise vectors $z_{n}\in R^{C}$ (n=1,…,N, N=B×D×H×W). Let the learnable codebook be $E=\{e_{k}{\}}_{k=1}^{K}\in R^{K\times C}$. Then, we compute the squared Euclidean distances as defined in Equation 1:

$$d_{nk}\;=\;\|z_{n}{\|}_{2}^{2}+\|e_{k}{\|}_{2}^{2}-2\;z_{n}^{⊤}e_{k},$$ (1)

The nearest code index is selected according to Equation 2:

$$k^{*}(n)=\arg\min_{k}\;d_{nk},$$ (2)

and form a one-hot assignment $y_{nk}=\|/-[k=k^{*}(n)]$. The quantized vector is given in Equation 3:

$${\hat{z}}_{n}=\sum_{k=1}^{K}y_{nk}\;e_{k}=E^{⊤}y_{n},$$ (3)

which is reshaped back to $\hat{Z}$ with the same spatial size as Z. A straight-through estimator is applied as shown in Equation 4:

$$\tilde{Z}=Z+(\hat{Z}-Z{)}_{\text{stop-grad}},$$ (4)

so the forward pass uses $\hat{Z}$ while gradients flow to Z. Then, Z needs to undergo a reshape operation to restore it feature shape. To prevent model collapse, a commitment constraints is utilized throughout training. Specifically, the optimization formula for the module is given in Equation 5:

$$L_{\text{VQ}}=\underset{\text{commitment, no grad on }\hat{Z}}{\underbrace{\|\hat{Z}-Z{\|}_{2}^{2}}}+\;\lambda \;\underset{\text{codebook update, no grad on }Z}{\underbrace{\|\hat{Z}-Z{\|}_{2}^{2}}}.$$ (5)

Here, the first term encourages the encoder outputs to stay close to their assigned codes, while the second term updates the codebook toward encoder outputs. λ is the weighting coefficients, and we take it as 0.25 based on the experimental results. In addition, we implement a codebook of K=64 learnable vectors (dimensionality C=64) to create a discrete latent space that is both highly compact and expressive, striking an effective balance between representation capacity and computational efficiency.

### 3.4 Global feature representation module

Map continuous latent features into discrete tokens to achieve discretization and compression of latent space. However, there is still a problem. The multi-scale context encoder typically employs 3D convolution operations to capture spatial contextual information within volumetric medical data during 3D feature extraction. However, in practice, such encoders tend to focus more on extracting 2D spatial features within individual slices, while under-modeling the cross-slice dependencies and longitudinal anatomical continuity between adjacent slices. This often neglects the intrinsic 3D structural correlations across different slices in CT sequences, potentially leading to inaccurate tumor boundary delineation, discontinuous organ segmentation, or missed detection of small lesions. Therefore, relying solely on local 2D features without effective modeling of global 3D contextual information limits the performance improvement of models in complex medical image segmentation tasks.

Here, to effectively model the long-range dependencies and three-dimensional anatomical continuity between different slices in the high-level semantic space, we have designed a global feature representation module. This module aims to address the limitations of traditional encoders in capturing cross-slice spatial correlations, particularly when processing 3D medical images. It enables better modeling of the structural consistency and morphological evolution of organs and lesions along the *z*-axis direction. As illustrated in Figure 2, we adopt the Mamba network as the core component of this module, replacing the Transformer architecture. Compared to Transformers based on self-attention mechanisms, Mamba leverages a Selective State Space Model (SSM) to efficiently model serialized features.

**Figure 4:**
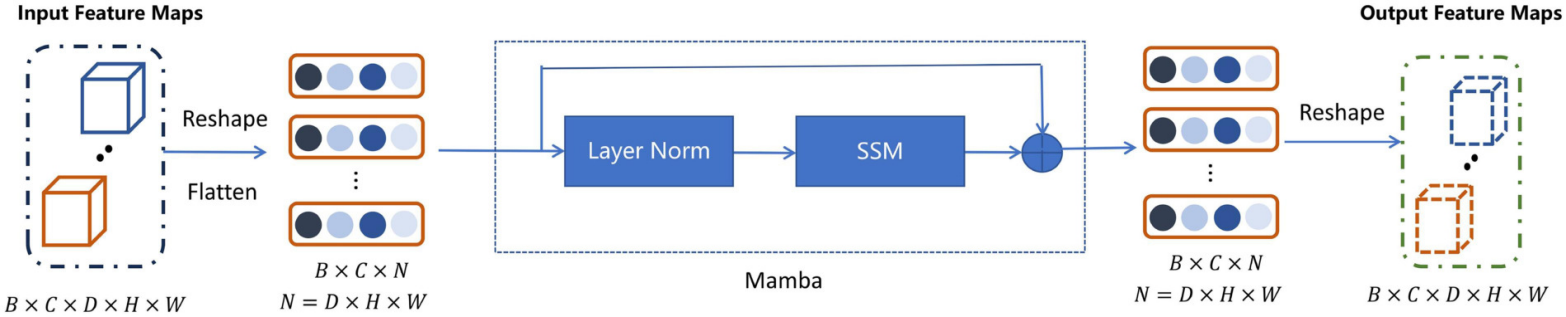
Visualization of Tensor Shape Transformation in the Mamba Module. The 3D feature volume is reshaped into a sequence for efficient sequential processing by Mamba, then reshaped back to 3D.

As shown in Figure 4, the input 3D feature map is flatten into a length sequence and feed it as a spatial sequence into the Mamba network. Formally, for an input sequence $x_{t}\in R^{d}$ at the depth position t, Mamba maintains a latent state $h_{t}\in R^{n}$ updated as defined in Equation 6:

$$h_{t}=\bar{A}(x_{t})h_{t-1}+\bar{B}(x_{t})x_{t},y_{t}=Ch_{t}$$ (6)

where $\bar{A}(x_{t})$ and $\bar{B}(x_{t})$ are input-dependent projection matrices generated by a linear layer, enabling the model to selectively propagate or suppress information. This mechanism allows Mamba to adaptively focus on anatomically relevant slices and ignore irrelevant or noisy ones. Here, the Mamba employs a state size of n=16 and operates on embeddings of dimension d=64. We placed this module at the bottleneck layer to enable Mamba to establish long-range, cross slice dependencies along the *z*-axis of volumetric data, effectively enhancing its ability to perceive overall organ morphology and spatial distribution of lesions.

### 3.5 The hybrid loss function

Incorporating prior knowledge during the model optimization phase can enhance the accuracy and robustness of medical image segmentation. Considering the anatomical characteristics of liver tumors, their spatial distribution follows a clear prior pattern—tumor lesions are entirely confined within the liver parenchyma and do not exist independently outside the liver tissue. Based on this, the liver region can be introduced as a spatial constraint prior during model training and inference.

Here, we propose an inclusiveness constraint $L_{\text{contain}}$ by jointly segmenting the liver and tumor regions, incorporating a constraint term into the loss function that enforces the tumor region to be entirely contained within the liver region. Integrating this prior knowledge not only effectively suppresses false positive predictions outside the liver boundary but also guides the network to focus more on learning lesion features within the liver, thereby improving the overall performance and clinical practicality of tumor segmentation. The specific computation is shown in Equation 7:

$$L_{\text{contain}}=\frac{1}{N}\sum_{x}\max(0,T(x)-L(x))$$ (7)

where T(x) and L(x) represent tumor and liver, respectively. If T(x)>L(x), it indicates that the model predicts the presence of a tumor at that location but the liver does not exist, which violates medical priors.

In summary, the overall objective combines a segmentation loss $L_{\text{seg}}$, the vector-quantization loss $L_{VQ}$, and consistent loss $L_{\mathrm{contain}}$ to optimize our method, as shown in Equation 8:

$$L=L_{\text{seg}}+\alpha \;L_{\text{VQ}}\;+\;\beta \;L_{\text{contain}}$$ (8)

where $L_{\text{contain}}$ is the constrained loss, α, and β are weighting coefficients.

## 4 Experiments

### 4.1 Datasets and preprocessing

The method was assessed on the Liver Tumor Segmentation Benchmark (LiTS) dataset [9], a publicly accessible benchmark introduced during the 2017 International Conference on Medical Image Computing and Computer-Assisted Intervention (MICCAI). This dataset was specifically curated to facilitate the evaluation and comparison of automated approaches for liver and liver tumor segmentation in contrast-enhanced abdominal CT volumes. It comprises 131 3D abdominal CT scans for training and 70 independent scans for testing, collected from diverse clinical centers and patients to ensure variability in imaging protocols and demographic characteristics. Each scan has a corresponding manual segmentation of the liver and liver tumors, performed by clinical experts. The training set includes annotations for both liver and tumor regions, while the test set annotations are withheld to enable blind evaluation. Due to the non-public nature of the test set, we randomly divided it into training set, validation set, and test set from the publicly available training set.

In the preprocessing stage, the voxel intensities of CT images were clipped to a fixed Hounsfield Unit (HU) range (−200, 200) to suppress noise and non-relevant intensity variations. The CT images were then resampled, applying cubic B-spline interpolation for images and nearest-neighbor interpolation for masks, with an in-plane downsampling factor of 0.5 and normalization of the *z*-axis spacing to 1 mm. Subsequently, the liver-containing 48 consecutive slices were randomly sampled along the axial direction to serve as network inputs. Finally, the CT volumes were normalized using min–max scaling to constrain the intensity range within standardized bounds.

### 4.2 Implementation details

All experiments were performed using PyTorch on an Ubuntu system with an NVIDIA RTX 4080 Super GPU equipped with 16 GB of memory. The proposed model was trained with the Adam optimizer using a batch size of 1 and a total of 160 epochs. The Adam optimizer was configured with parameters: initial learning rate of $1\times 10^{-4}$, $\beta_{1}=0.9$, $\beta_{2}=0.999$, $\varepsilon =1\times 10^{-8}$, and $\text{weight\_decay}=1\times 10^{-5}$. To ensure statistical robustness, all experiments were repeated three times using fixed random seeds (42, 57, and 89). The reported results represent the mean ± standard deviation across these three runs. This protocol was applied consistently to all models, including baselines and ablations, under identical hyperparameter settings and data splits.

### 4.3 Evaluation metrics

To assess the effectiveness of our proposed method, we followed the evaluation protocol of the LiTS Challenge and used the Dice score (DICE), Volume Overlap Error (VOE), Relative Volume Difference (RVD), Average Symmetric Surface Distance (ASD), and 95th percentile Hausdorff Distance (95HD) as key metrics for evaluating segmentation accuracy. The definitions of these metrics are provided below in Equations 9–13:

$$\mathrm{DICE}(P,G)=\frac{2|P⋂G|}{\left|P|+|G\right|}$$ (9)

$$\mathrm{VOE}(P,G)=1-\frac{2|P⋂G|}{\left|P⋃G\right|}$$ (10)

$$\mathrm{RVD}(P,G)=\frac{\left|P|-|G\right|}{\left|P\right|}$$ (11)

$$\mathrm{ASD}(P,G)=\frac{1}{\left|\partial P|+|\partial G\right|}\left(\sum_{\;p\in \partial P}d(p,\partial G)+\sum_{g\in \partial G}d(g,\partial P)\right)$$ (12)

95HD(P,G)=percentile({d(p,∂G)∣p∈∂P}∪{d(g,∂P)∣g∈∂G},95) (13)

where P represents the prediction result of the network, G represents the ground truth, respectively.

## 5 Results and discussion

### 5.1 Ablation experiment

To assess the contribution of each proposed component, we conducted a series of ablation studies to analyze their impact on performance. Specifically, we adopted a 3D U-Net with a four-layer encoder-decoder structure as the baseline, and incrementally incorporated the proposed modules to quantitatively evaluate their effectiveness. Furthermore, we examined the influence of the mixed loss function by progressively introducing additional loss terms into the overall objective. In addition, we focused on evaluating the impact of different parameters in the CodeBook module on model performance. All experiments were conducted under identical settings to ensure fair and consistent comparison. It should be pointed out that due to subtle differences in ablation, we report the results using more refined numbers.

Table 1 shows the experimental results of component ablation. We can observe that nearly all the proposed modules contribute positively to the performance. Specifically, adding the multi-scale context encoder (MCE) module to the baseline improves the accuracy of all metrics. Notably, for liver segmentation, the ASD and 95HD metrics are reduced by 0.247 and 0.938 mm, indicating that the introduction of the MCE module effectively enhances the geometric consistency and boundary precision by capturing tumor characteristics across different spatial resolutions. When the CodeBook (CB) module is further integrated into the framework, additional improvements are observed in segmentation. The CB module can effectively capture and model the underlying patterns of liver tumors in terms of spatial distribution and morphological structure through learnable semantic prototypes, thereby improving segmentation accuracy and suppressing feature redundancy. Finally, we integrated the global feature representation (GFR) module into the model and observed that it significantly improves nearly all evaluation metrics for both organs. The enhanced performance confirms that strengthening global relational learning via the Mamba and fusing multi-scale contextual features can effectively boost the accuracy and robustness of organ segmentation, particularly under challenging conditions such as lesion heterogeneity and ambiguous boundaries.

**Table 1:** Component ablation results in liver and tumor segmentation.

Organ	Model	DICE ↑	VOE ↓	RVD ↓	ASD ↓	95HD ↓
Liver	Baseline	0.9067 ± 0.0340	0.0933 ± 0.0340	0.1200 ± 0.0394	2.3353 ± 0.2258	10.7045 ± 0.9941
	Model1	0.9263 ± 0.0102	0.0692 ± 0.0070	0.0681 ± 0.0217	2.0325 ± 0.4048	9.3952 ± 2.7491
	Model2	0.9295 ± 0.0032	0.0672 ± 0.0035	0.0650 ± 0.0125	1.9486 ± 0.0491	8.8617 ± 0.1104
	Model3	0.9363 ± 0.0076	0.0637 ± 0.0076	0.0636 ± 0.0151	1.9365 ± 0.0910	8.8068 ± 1.0373
Tumor	Baseline	0.4500 ± 0.0254	0.5367 ± 0.0086	0.7199 ± 0.1560	14.7240 ± 1.9682	35.5782 ± 0.9416
	Model1	0.4521 ± 0.0049	0.5350 ± 0.0650	0.7186 ± 0.0234	13.7522 ± 4.0486	35.3641 ± 4.5694
	Model2	0.4555 ± 0.0672	0.5348 ± 0.0789	0.6539 ± 0.0177	12.9723 ± 0.8568	35.1449 ± 4.0067
	Model3	0.4588 ± 0.0627	0.5347 ± 0.0454	0.6572 ± 0.0396	13.0150 ± 0.8691	35.1284 ± 10.0678

Model1: Baseline+MCE, Model2: Baseline+MCE+CB, Model3: Baseline+MCE+CB+GFR.

**Figure 5:**
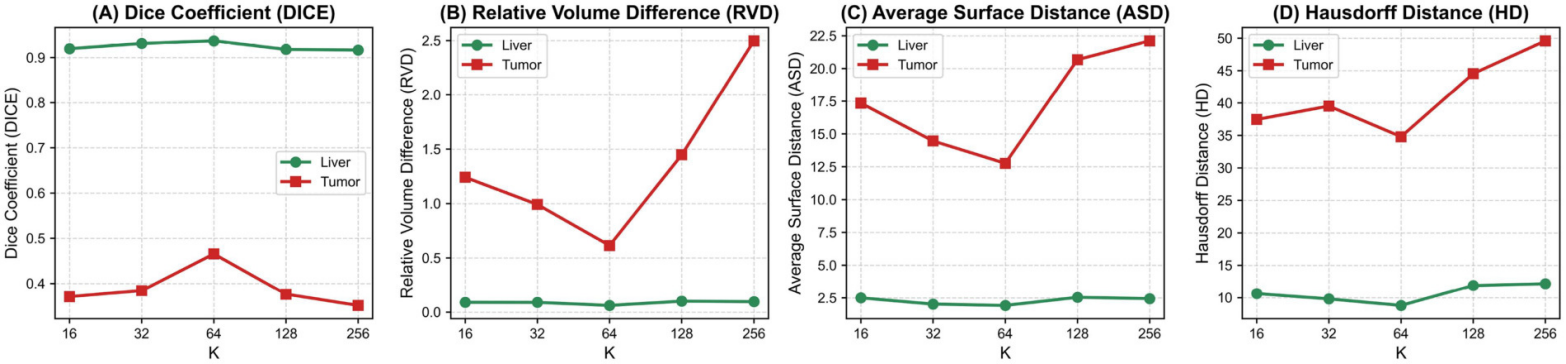
Performance sensitivity of codebook size K. The *x*-axis represents the number of learnable prototypes (K) in the codebook module. Each subplot illustrates the impact of varying K on a specific segmentation metric for both liver (green line) and tumor (red line) segmentation. Display the results of DICE **(A)**, RVD **(B)**, ASD **(C)** and 95HD **(D)**.

To further investigate the sensitivity of the CodeBook module to its key hyperparameters, we conduct a systematic ablation study by varying the codebook size K, and loss weights (λ for commitment loss). All experiments are performed on our proposed model under identical training and evaluation settings. First, we evaluate the impact of codebook capacity by testing different values of K∈{16,32,64,128,256}. As shown in Figure 5, increasing K initially improves segmentation accuracy (e.g., DICE, RVD, ASD), suggesting that a larger codebook can capture more diverse tissue patterns. However, performance plateaus when K>64, with marginal gains and increased memory overhead, indicating diminishing returns in representational benefit.

Then, we analyze the effect of λ∈{0.10,0.20,0.25,0.30,0.35}, which serves as the loss weight for the commitment loss term within the CodeBook module. This parameter balances the contribution of the commitment loss $L_{\text{commit}}$ relative to the primary segmentation loss. As shown in Table 2, setting λ too low (e.g., 0.10) results in insufficient commitment pressure, leading to poor utilization of the learned codebook prototypes and suboptimal feature quantization. Conversely, an overly high value (e.g., 0.35) overly constrains the encoder’s feature learning, potentially degrading segmentation accuracy as the encoder prioritizes matching codebook entries over producing accurate segmentation maps. An optimal value of λ=0.25 provides the best trade-off, ensuring effective prototype learning and quantization while maintaining high segmentation fidelity. This sensitivity analysis confirms that careful tuning of the commitment loss weight is essential for maximizing the benefits of the CodeBook module.

**Table 2:** Experimental results of liver and tumor segmentation using different λ coefficients.

Organ	λ	DICE ↑	VOE ↓	RVD ↓	ASD ↓	95HD ↓
Liver	0.10	0.9209 ± 0.0105	0.0791 ± 0.0105	0.0962 ± 0.0104	2.5126 ± 0.7017	12.0698 ± 3.9518
	0.20	0.9202 ± 0.0171	0.0798 ± 0.0171	0.0962 ± 0.0210	2.4205 ± 0.1815	11.5994 ± 2.0632
	0.25	0.9370 ± 0.0006	0.0630 ± 0.0006	0.0609 ± 0.0036	1.9238 ± 0.2828	8.7884 ± 2.2282
	0.30	0.8919 ± 0.0427	0.1081 ± 0.0427	0.1317 ± 0.0414	3.3580 ± 0.3589	13.7055 ± 3.1622
	0.35	0.9248 ± 0.0006	0.0097 ± 0.0097	0.0896 ± 0.0134	2.5768 ± 0.9113	12.8719 ± 6.4655
Tumor	0.10	0.3751 ± 0.0302	0.6249 ± 0.0302	1.0556 ± 0.4728	19.3859 ± 3.9737	40.9398 ± 2.2204
	0.20	0.3761 ± 0.0539	0.6239 ± 0.0539	1.5466 ± 0.4336	19.2929 ± 4.0996	38.6371 ± 6.5570
	0.25	0.4655 ± 0.0672	0.5345 ± 0.0672	0.6139 ± 0.0529	12.7640 ± 3.4228	34.8104 ± 6.2128
	0.30	0.3838 ± 0.0306	0.6162 ± 0.0306	0.6707 ± 0.1731	17.8277 ± 1.9927	38.3287 ± 4.6225
	0.35	0.3728 ± 0.0282	0.6272 ± 0.0282	1.6449 ± 1.5598	19.2230 ± 2.9567	42.9577 ± 4.2730

**Figure 6:**
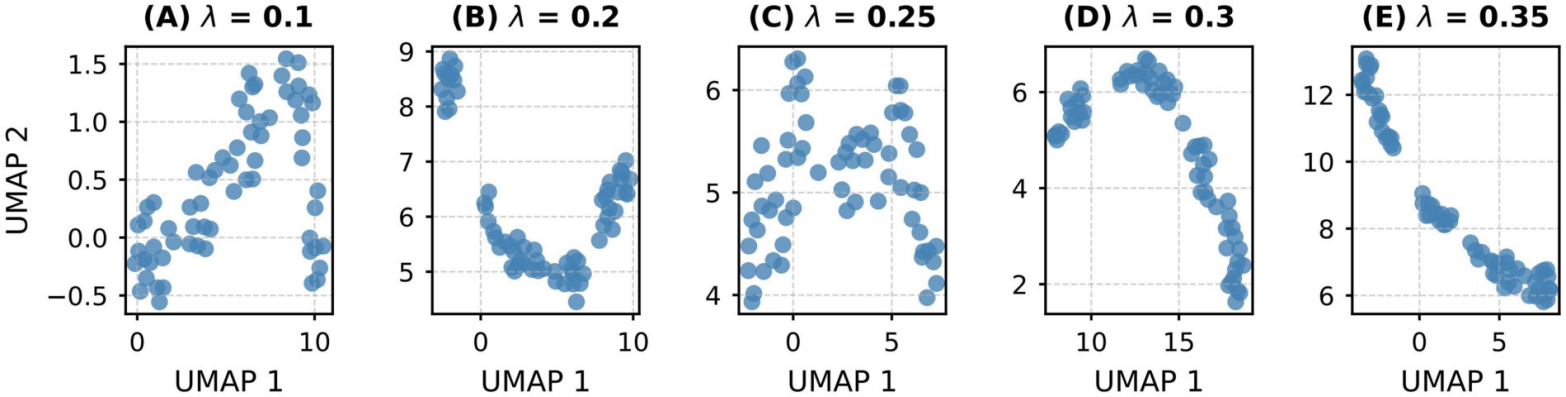
Visualization of learned codebook prototypes in 2D UMAP space under varying regularization strength λ. Each subplot displays the spatial distribution of K=64 semantic prototypes derived from high-dimensional feature embeddings, with **(A)**–**(E)** corresponding to λ=0.1,0.2,0.25,0.3,0.35, respectively.

Finally, we visualize the K=64 codebook prototypes projected onto a 2D UMAP manifold for different values of the regularization weight λ. As shown in Figure 6, the geometric organization of these prototypes varies significantly with λ. At low λ (e.g., 0.1 and 0.2), the prototypes are densely clustered, indicating excessive quantization pressure that collapses semantically distinct features into overly compact regions, a phenomenon that may lead to loss of discriminative power, particularly for small or heterogeneous tumors. Conversely, at high λ (e.g., 0.3 and 0.35), the prototypes become increasingly dispersed and elongated, suggesting insufficient regularization that fails to enforce feature coherence, potentially reintroducing redundancy and noise sensitivity. Critically, at λ=0.25, the prototype distribution achieves an optimal balance: clusters are neither too tight nor too diffuse. This intermediate configuration reflects a well-calibrated regularization regime that encourages the network to learn discriminative yet compact representations. These representations are dense enough to suppress irrelevant variation while being diverse enough to preserve critical anatomical distinctions.

To quantitatively assess the efficacy of the Mamba in modeling long-range dependencies, we conduct an ablation study by replacing the Mamba block with a standard Multi-Head Self-Attention (MHSA) [48] that shares identical input and output dimensionalities. We compare the two architectures in terms of total trainable parameters, GPU memory consumption during inference, and per-sample inference latency. All experiments are performed on the same hardware (NVIDIA RTX4080 Super GPU) and under identical input configurations to ensure a fair comparison. As shown in Table 3, the input tensor is fixed at 64×1×8×8, which is the output result from the CodeBook module. Under batch size=1, Mamba achieves a significantly faster inference time of 91 μs compared to MHSA’s 193 μs, while consuming slightly less GPU memory (8.24 M vs. 8.36 M). This indicates that Mamba is more computationally efficient for single-sample inference. When the batch size increases to 10, both models exhibit longer inference times due to increased computational load. However, Mamba still maintains a clear advantage with 96 μs per sample, whereas MHSA requires 320 μs per sample. Regarding memory consumption, both models show a modest increase from batch size 1 to 10: Mamba grows from 8.24 M to 8.66 M, and MHSA from 8.36 M to 8.74 M. The memory overhead introduced by larger batches is minimal, suggesting good memory scalability for both architectures. In summary, Mamba demonstrates superior inference speed across both batch sizes while maintaining competitive memory usage, making it a more suitable choice for real-time or resource-constrained applications.

**Table 3:** Inference efficiency comparison of Mamba and MHSA models under different batch sizes.

			Batch size=1		Batch size=10	
Input tensor	Model	Params	Inference time	GPU memory	Inference time	GPU memory
64×1×8×8	Mamba	16,640	0.000091	8.24 M	0.000096	8.66 M
	MHSA	32,768	0.000193	8.36 M	0.000320	8.74 M

Table 4 summarizes the ablation study results assessing the effectiveness of the proposed hybrid loss function. The baseline model here is our proposed method, which only includes segment loss $L_{\text{seg}}$. As shown, performance progressively improves with the incremental addition of each loss component. For liver segmentation, Model1 (using only $L_{\text{seg}}$) achieves a DICE of 0.9159, establishing a strong baseline. Adding $L_{\text{VQ}}$ (Model2) improves all metrics, indicating that vector quantization enhances feature discriminability and spatial coherence. Further incorporating $L_{\text{contain}}$ (Model3) yields the highest DICE (0.9370) and lowest 95HD (8.79), demonstrating its critical role in sharpening boundaries. Similar gains are observed for tumor segmentation. Together, the hybrid loss jointly optimizes segmentation accuracy, codebook representation, and boundary geometry, synergistically boosting both global and local performance.

**Table 4:** Experimental results of loss function ablation on liver and tumor segmentation.

Organ	Model	DICE ↑	VOE ↓	RVD ↓	ASD ↓	95HD ↓
Liver	Model1	0.9159 ± 0.0068	0.0841 ± 0.0068	0.0946 ± 0.0179	2.3166 ± 0.2985	11.1110 ± 3.0907
	Model2	0.9363 ± 0.0076	0.0637 ± 0.0076	0.0636 ± 0.0151	1.9365 ± 0.0910	8.8068 ± 1.0373
	Model3	0.9370 ± 0.0006	0.0630 ± 0.0006	0.0609 ± 0.0036	1.9238 ± 0.2828	8.7884 ± 2.2282
Tumor	Model1	0.3849 ± 0.0145	0.6151 ± 0.0145	0.8878 ± 0.3602	16.4598 ± 2.5372	38.7509 ± 1.6418
	Model2	0.4588 ± 0.0627	0.5347 ± 0.0454	0.6572 ± 0.0396	13.0150 ± 0.8691	35.1284 ± 10.0678
	Model3	0.4655 ± 0.0672	0.5345 ± 0.0672	0.6139 ± 0.0529	12.7640 ± 3.4228	34.8104 ± 6.2128

Model1: Our Method (only $L_{\text{seg}}$), Model2: Our Method+$L_{\text{VQ}}$, Model3: Our Method+$L_{\text{VQ}}$+$L_{\text{contain}}$.

**Figure 7:**
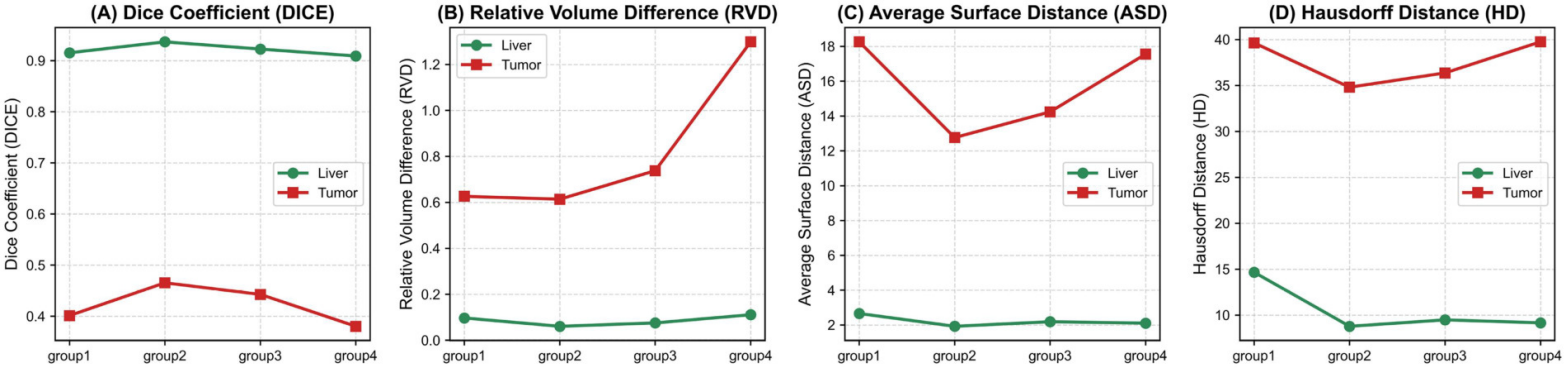
Sensitivity analysis of loss weights (α,β) on liver and tumor segmentation performance. Group1–4 correspond to (α,β)=(0.2,0.8),(0.4,0.6),(0.6,0.4),(0.8,0.2), respectively. Metrics: **(A)** DICE, **(B)** RVD, **(C)** ASD, **(D)** HD.

Since different values of α and β determine the optimization direction of the model, we further explored the optimal settings for these coefficients. The experimental results are shown in Figure 7. It should be noted that in Figure 7, Group 1 represents α=0.2, β=0.8; Group 2 represents α=0.4, β = 0.6; Group 3 represents α = 0.6, β = 0.4; and Group 4 represents α = 0.8, β = 0.2. For liver segmentation, all metrics remain relatively stable across groups. This suggests that liver segmentation is robust to weight variations, likely due to its larger size and more consistent anatomical structure. In contrast, tumor segmentation exhibits strong sensitivity to the loss weights. Notably, as β decreases, tumor boundary errors (ASD, HD) increase sharply, indicating that insufficient emphasis on the containment constraint $L_{\text{contain}}$ leads to inaccurate tumor localization, especially near liver boundaries. This demonstrates a critical trade-off: while increasing α strengthens feature representation via codebook regularization, over-prioritizing it compromises the anatomical constraint that tumors must lie within the liver. The optimal balance is achieved at Group2 (α=0.4,β=0.6), where both global accuracy and local boundary precision are maximized for tumors.

### 5.2 Comparison experiment

To further validate the effectiveness of the proposed method, we conducted a comparative analysis with several widely-used 3D medical image segmentation frameworks, including 3D U-Net [47], VNet [49], Att-UNet [50], SegResNet [51], ResUnet [52], UNETR [43], CodeBookUNet, and VAEUNet [53]. For a fair and consistent evaluation, all methods were subjected to the same preprocessing steps, and identical training, validation, and test splits were used during result reproduction. Specifically, 3D U-Net [47], VAEUnet [53], CodebokUnet, and ResUnet [52] are the networks we replicated, while SegResNet [51], VNet [49], Att-UNet [50], and UNTR [43] are derived from the MONAI framework [54].

Table 5 presents a comprehensive quantitative comparison of liver and tumor segmentation performance across several widely-used 3D medical image segmentation framework. All metrics are reported as mean ± standard deviation over three different seed points. Generally speaking, our proposed network achieves superior performance on both liver and tumor segmentation tasks across all evaluated metrics on the LiTS dataset. For liver segmentation, our method attains the highest Dice coefficient, lowest VOE, RVD, ASD, and 95HD. These results significantly outperform strong baselines such as CodeBookUNet (Dice: 0.93) and VAEUNet [53] (Dice: 0.92), particularly in boundary precision (ASD and 95HD), indicating superior anatomical fidelity. For tumor segmentation, a more challenging task due to lesion heterogeneity and small size, our method again leads with a Dice of 0.47 ± 0.07, notably higher than UNETR [43] (0.41) and VAEUNet [53] (0.38), while also achieving the lowest volumetric error (RVD=0.61 ± 0.05) and spatial discrepancy (ASD=12.76 ± 3.42, 95HD=34.81 ± 6.21). This consistent superiority across both global overlap and local boundary metrics demonstrates the robustness of our approach under complex clinical conditions.

**Table 5:** Performance comparison on liver and tumor segmentation.

Organ	Model	DICE ↑	VOE ↓	RVD ↓	ASD ↓	95HD ↓
Liver	3D U-Net	0.91 ± 0.03	0.09 ± 0.03	0.12 ± 0.04	2.33 ± 0.23	10.70 ± 0.99
	VNet	0.83 ± 0.07	0.17 ± 0.07	0.18 ± 0.07	4.42 ± 1.64	14.73 ± 2.02
	Att-UNet	0.89 ± 0.00	0.11 ± 0.00	0.11 ± 0.01	4.73 ± 0.63	25.53 ± 1.22
	SegResNet	0.93 ± 0.01	0.07 ± 0.00	0.07 ± 0.03	2.20 ± 0.69	11.22 ± 4.41
	ResUnet	0.92 ± 0.00	0.08 ± 0.00	0.07 ± 0.03	3.27 ± 0.74	16.09 ± 4.08
	UNETR	0.91 ± 0.01	0.09 ± 0.01	0.12 ± 0.05	4.56 ± 1.58	18.78 ± 3.06
	CodeBookUNet	0.93 ± 0.01	0.07 ± 0.01	0.09 ± 0.02	1.61 ± 0.34	7.07 ± 1.88
	VAEUNet	0.92 ± 0.01	0.08 ± 0.01	0.08 ± 0.03	2.20 ± 0.21	10.82 ± 0.96
	Our method	0.94 ± 0.00	0.06 ± 0.00	0.06 ± 0.00	1.92 ± 0.28	8.79 ± 2.23
Tumor	3D U-Net	0.45 ± 0.03	0.54 ± 0.01	0.72 ± 0.16	14.72 ± 1.97	35.58 ± 0.94
	VNet	0.45 ± 0.03	0.55 ± 0.02	0.69 ± 0.13	14.85 ± 6.74	37.44 ± 12.79
	Att-UNet	0.46 ± 0.05	0.54 ± 0.05	1.40 ± 0.22	19.74 ± 9.91	43.97 ± 19.01
	SegResNet	0.45 ± 0.06	0.55 ± 0.06	0.64 ± 0.09	14.23 ± 1.25	35.48 ± 0.91
	ResUnet	0.41 ± 0.04	0.59 ± 0.04	1.79 ± 0.71	18.79 ± 0.81	40.51 ± 4.67
	UNETR	0.31 ± 0.04	0.69 ± 0.04	3.33 ± 6.82	24.89 ± 1.23	51.72 ± 4.62
	CodeBookUNet	0.42 ± 0.06	0.58 ± 0.06	0.73±0.15	14.00 ± 5.84	35.10 ± 10.12
	VAEUNet	0.38 ± 0.01	0.62 ± 0.01	0.91±0.46	17.98 ± 6.42	36.30 ± 5.57
	Our method	0.47 ± 0.07	0.53 ± 0.07	0.61 ± 0.05	12.76 ± 3.42	34.81 ± 6.21

A standard deviation of 0 indicates that the value is too small.

The superior performance of our method stems directly from its architectural innovations designed to address the core challenges outlined in our motivation. First, the multi-scale texture encoder effectively captures heterogeneous tumor patterns at different resolutions, mitigating the sensitivity of standard CNNs to scale variation. Second, the learnable codebook module promotes compact, discriminative feature representations by quantizing redundant activations into semantic prototypes, enhancing generalization and reducing overfitting to noisy or ambiguous regions, a common pitfall for methods like SegResNet [51] and Att-UNet [50] that rely solely on dense feature maps. Third, the Mamba-based global relational module enables efficient long-range context modeling across 3D slices. This capability is critical for tumors that span multiple axial planes. Traditional attention mechanisms, such as those in UNETR [43], struggle to capture such long-range dependencies. Local convolutional operations, like those in 3D U-Net [47], also face difficulties. They often require excessive computational cost or suffer from information loss. Finally, the explicit incorporation of anatomical prior through inclusion loss ensures biologically plausible outputs. This prevents the generation of false-positive tumor predictions outside the liver, an issue frequently observed in transformer-based models like UNETR [43] that lack such structural constraints.

In contrast, many existing methods exhibit degraded performance due to their inability to simultaneously model multi-scale features, global dependencies, and anatomical plausibility. For instance, while UNETR [43] leverages global self-attention, it often produces fragmented boundaries (high 95HD) due to insufficient spatial regularization; similarly, VAEUNet [53] contains the probabilistic framework may introduce uncertainty that degrades localization accuracy. Our integrated design to some extent collaboratively addresses these limitations, providing a more accurate and clinically significant solution. The ablation experiment also demonstrated the effectiveness of the module.

**Figure 8:**
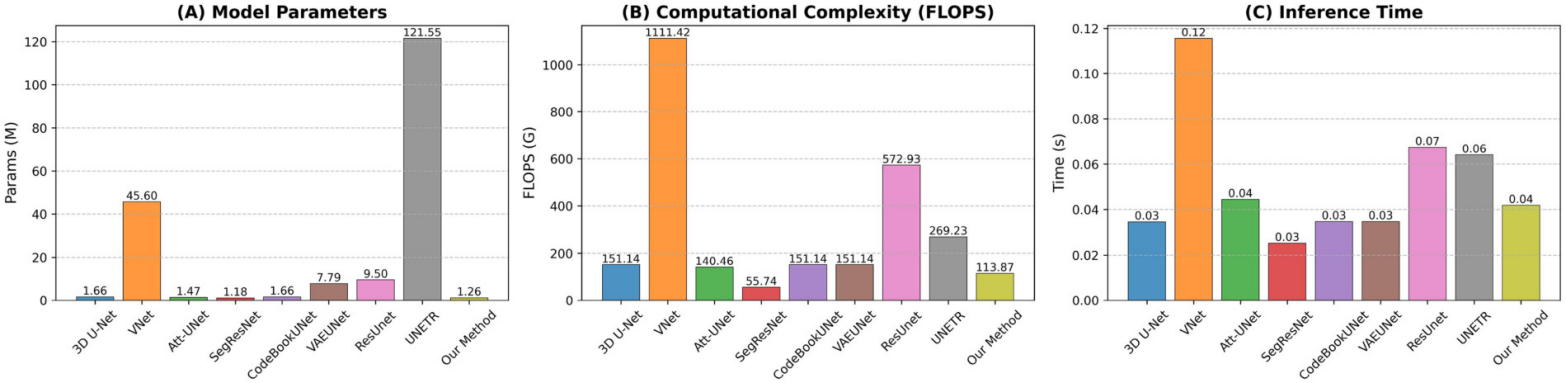
Efficiency comparison of segmentation models on a unified platform: **(A)** model parameters (M), **(B)** computational complexity (FLOPS, G), and **(C)** inference time (s). Our method achieves the lowest parameter count and competitive inference speed while maintaining moderate computational cost.

Figure 8 compares model efficiency under identical conditions. Our method achieves the lowest parameter count (1.26 M), even below SegResNet and Att-UNet, enabling deployment on resource-constrained systems. With 113.87G FLOPS, it matches 3D U-Net and CodeBookUNet while being far lighter than VNet and UNETR. Crucially, its inference time (0.04 s) matches or exceeds leading lightweight models, including SegResNet and 3D U-Net. Together, these results demonstrate that our method delivers the best trade-off: minimal size, moderate computation, and fast inference, ideal for real-time medical segmentation.

**Figure 9:**
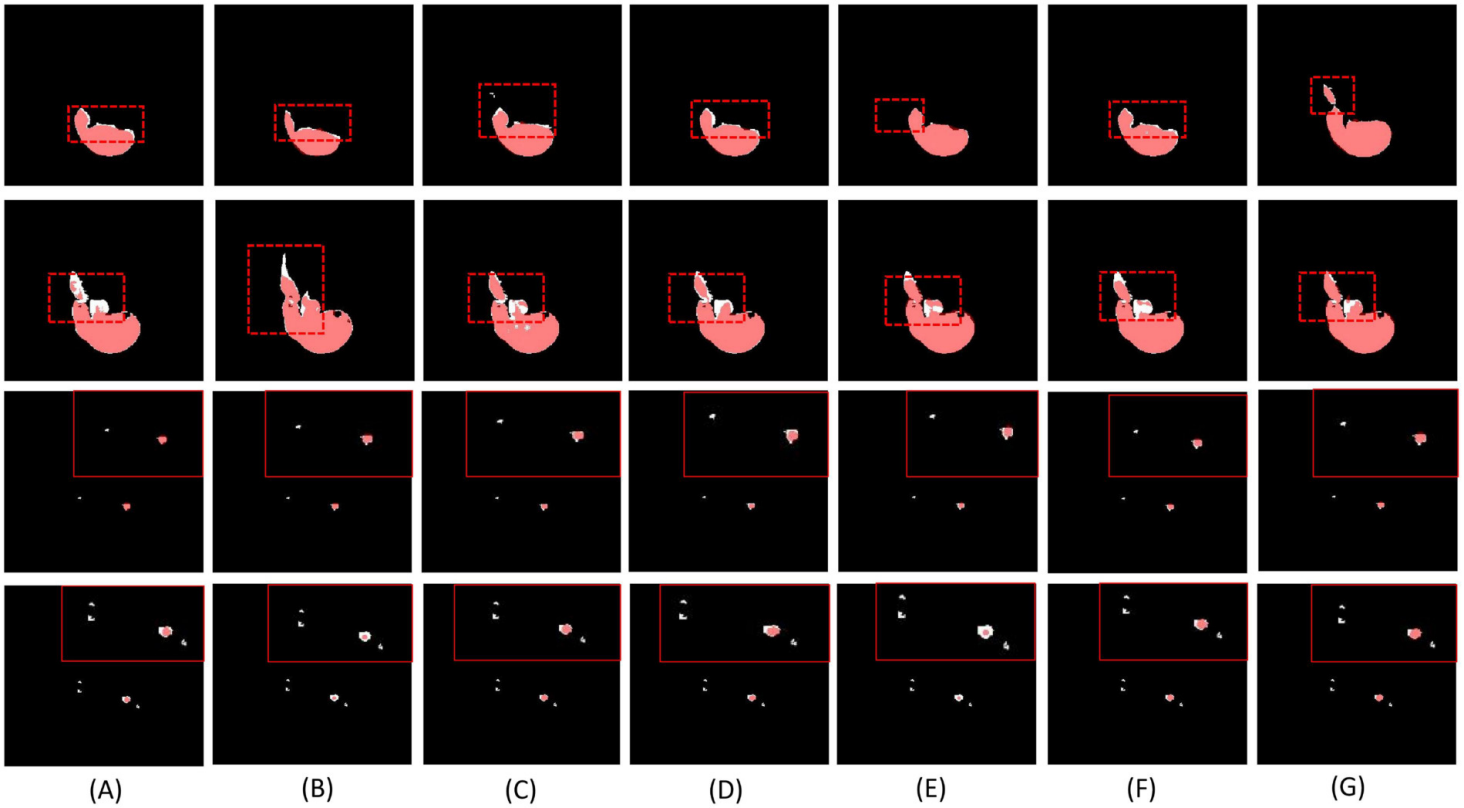
The visual results of liver segmentation, where red represents predicted pixels and white represents annotated results. The upper two lines show the liver segmentation results, while the lower two lines show the tumor segmentation results. **(A)** 3D U-Net [47]. **(B)** VNet [49]. **(C)** Att-UNet [50]. **(D)** SegResNet [51]. **(E)** CodeBookUNet. **(F)** VAEUNet [53]. **(G)** Our Method.

Figure 9 shows the liver segmentation outcomes, where (A) to (G) correspond to the segmentation results produced by the methods or models listed in Table 1 on the same dataset. The upper two lines show the liver, while the lower two lines show the tumor segmentation results. (A) and (B) exhibit coarse boundaries with obvious under-segmentation and false positives, while (C) to (F) show progressive improvements, yet still lack detail in complex regions. In contrast, our method (G) achieve clear, accurate boundaries, well-preserved structural integrity, and minimal errors.The results show that our model surpasses competing methods in both accuracy and consistency, providing robust performance that holds promise for clinical applications in liver segmentation.

### 5.3 Robust analysis

**Figure 10:**
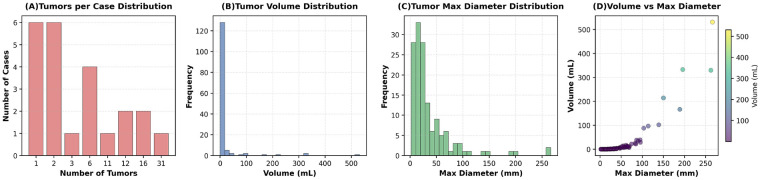
Statistical analysis of tumor characteristics in the test set: **(A)** distribution of tumor count per case, **(B)** tumor volume distribution, **(C)** maximum diameter distribution, and **(D)** scatter plot of tumor volume vs. maximum diameter.

The test set exhibits substantial heterogeneity in tumor size and multiplicity, as shown in Figure 10. While most cases contain 1–2 tumors, tumor volumes range from near-zero to over 500 mL, with maximum diameters spanning 10–250 mm. Notably, larger tumors tend to have higher volumes but with high variability, indicating that tumor morphology is not uniformly scaled. we further evaluate how different segmentation models perform across tumors of varying sizes. In clinical practice, accurate delineation of both small lesions (e.g., <20 mL) and large masses (e.g., >200 mL) is equally critical, yet many models struggle with extreme sizes due to scale mismatch or insufficient receptive field. By stratifying performance by tumor volume or diameter, we can identify whether a model generalizes well across the full spectrum of tumor morphologies present in real-world data.

Specifically,we stratify test tumors into five size categories based on maximum voxel diameter: small (<10 voxels), small-medium (10–20 voxels), medium (20–30 voxels), large (30–50 voxels), and very large (>50 voxels). Given the clinical relevance and inherent challenges of segmenting very small lesions, which are often overlooked or segmented by traditional models, we will focus quantitative analysis on the DICE metric. These metrics jointly capture segmentation accuracy, boundary precision, and lesion completeness, enabling a nuanced assessment of performance degradation at the extreme small-end of the size spectrum. The results across size strata reveal how each model generalizes under real-world conditions where tumor morphology varies dramatically.

**Figure 11:**
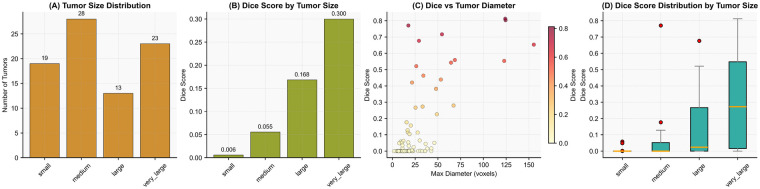
Model robustness analysis across tumor sizes: **(A)** distribution of tumor counts per size category, **(B)** mean DICE score by tumor size, **(C)** scatter plot of DICE vs. maximum tumor diameter (voxels), and **(D)** box plot of DICE score distribution per size group.

As shown in Figure 11A, the test set contains a balanced distribution of tumor sizes, with medium and very large tumors being most prevalent. DICE scores exhibit a clear positive correlation with tumor size (Figures 11B–D). While models achieve near-perfect performance on very large tumors, performance drops sharply for small tumors, with mean DICE falling below 0.05. The scatter plot in Figure 11C confirms this trend: as tumor diameter increases, DICE scores rise monotonically, though with high variance at smaller diameters. Box plots in Figure 11D further reveal that small tumors not only have low median DICE but also exhibit extreme outliers, indicating frequent complete failure to detect or segment these lesions. This analysis reveals a critical limitation of current segmentation architectures. They often fail to reliably detect small tumors. Yet, small tumors represent a significant portion of clinical cases. Our results emphasize the need for models with greater sensitivity to fine-scale structures. Improving this capability is a key direction for future work in medical image segmentation.

### 5.4 Limitations and future work

This study evaluates the proposed method on the widely used LiTS CT dataset, following standardized preprocessing and fair comparison protocols. While LiTS is a representative multi-center benchmark, validation is still mainly limited to a single public dataset. Future work will further test the method on additional independent multi-center cohorts to better assess generalization across scanners, acquisition protocols, and patient populations, and to facilitate real-world clinical deployment.

Our size-stratified analysis also indicates a clear limitation for extremely small tumors (few-voxel lesions), where miss-detections occur under severe class imbalance and Dice becomes highly sensitive to minor voxel errors, partial-volume effects, and annotation uncertainty. Although the multi-scale encoder contains local receptive-field branches, it is not explicitly optimized for ultra-small lesions. Future work will incorporate small-lesion–aware strategies to improve sensitivity for tiny tumors.

Finally, the codebook module contributes to representation regularization, yet its medical interpretability remains limited under the current supervision because LiTS lacks subregion-level annotations (e.g., boundary/necrosis/edema), so a direct mapping from codewords to clinically meaningful tissue semantics cannot be guaranteed. Moreover, our results suggest that higher feature flexibility is not always better. Achieving robust medical segmentation requires balancing expressive power with structural priors and constraints. Future work will adopt clinically grounded interpretability protocols and leverage datasets with richer annotations to better relate prototypes to anatomical/pathological concepts, and explore intermediate codebook designs to better trade off flexibility and generalization.

## 6 Conclusion

In this study, we present a novel deep learning framework for joint liver and tumor segmentation in 3D CT images. By integrating a multi-scale texture encoder, a Mamba-based global relational module, and a compact codebook learning mechanism, our model effectively captures both local texture variations and long-range spatial dependencies. The introduction of anatomical prior through an inclusion loss further enhances the biological plausibility and accuracy of the predictions. Experimental results on the benchmark LiTS dataset demonstrate that our method achieves superior performance compared to existing state-of-the-art approaches, particularly in challenging cases involving small or heterogeneous tumors. The proposed architecture provides a robust and generalizable solution for 3D medical image segmentation, with potential applications in computer-aided diagnosis and surgical planning. Future work will focus on extending the framework to multi-institutional datasets and exploring dynamic codebook mechanisms for improved adaptability.

## Data availability statement

The original contributions presented in the study are included in the article/Supplementary Material, further inquiries can be directed to the corresponding author.
